# Lemierre syndrome complicating otitis media caused by *Streptococcus pneumoniae*

**DOI:** 10.1016/j.idcr.2022.e01382

**Published:** 2022-01-06

**Authors:** Abdulrahman F. Al-Mashdali, Akram F. Al-Warqi

**Affiliations:** a Internal Medicine Department, Hamad Medical Corporation, Doha, Qatar; b Clinical Radiology Department, Hamad Medical Corporation, Doha, Qatar

**Keywords:** Lemierre syndrome, Streptococcus pneumonia, Otitis media, Internal jugular vein thrombosis

A 34-year-old Asian male presented to our emergency department with fever, vomiting, and right ear pain for three days. He also reported ear discharge and right-sided neck pain. His past medical history was significant for extraskeletal Ewing sarcoma of gluteus muscle on chemotherapy (VAC protocol:Vincristine/Doxorubicin/Cyclophosphamide)and type 2 diabetes mellitus.On examination, he was febrile, and he had right-sided facial nerve palsy. Laboratory results were significant for elevated inflammatory markers. Computed tomography of temporal bone showed acute otitis media (AOM) features with filling defect in the right internal jugular vein. He was seen by an otolaryngologist who confirmed the diagnosis of AOM. Blood culture grew *Streptococcus pneumoniae*. The patient was started on intravenous ceftriaxone (2 g daily), and the fever subsided within 48 h. Magnetic resonance imaging (MRI) confirmed the diagnosis of right-sided labyrinthitis with secondary involvement of the right facial nerve and right jugular bulb thrombus (Fig. 1). Given the presence of otitis media, streptococcal bacteremia, and internal jugular vein thrombosis, Lemierre syndrome was diagnosed. On day 7, the patient was discharged with an intravenous ceftriaxone prescription for four weeks.

**Fig. 1:**
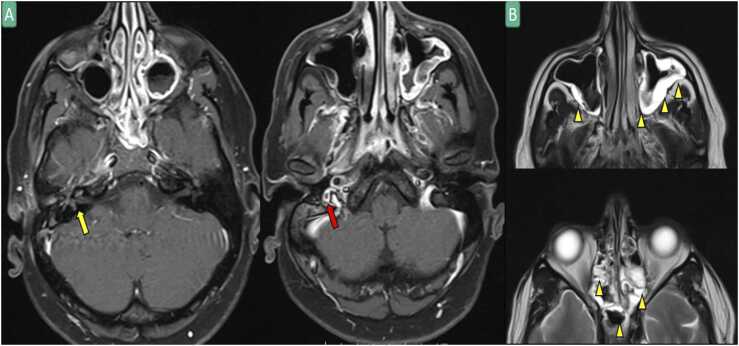
Axial brain T1WI post-contrast with fat saturation (A) showing evidence of enhancement at the right membranous labyrinth, cochlea, vestibule, semi-circular canals, and facial nerve (yellow arrow) as compared with the contralateral side as well as a small lateral eccentric filling defect at the right jugular bulb (red arrow) suggesting thrombosis. Selected axial T2WI (B) shows diffuse polypoid mucosal thickening involving the bilateral maxillary, sphenoidal sinuses, and ethmoid air cells (yellow arrowhead).

Lemierre syndrome is an infrequent condition, typically occurring in healthy young adults, characterized by septic thrombophlebitis of the internal jugular vein (IJV), and often associated with bacteremia and septic emboli to different organs, especially the lung. Lemierre syndrome usually complicates pharyngitis/tonsillitis, and less commonly otitis media or mastoiditis, as in our case [1]. Fusobacterium necrophorum is the most common isolated organism from blood culture in patients with Lemierre syndrome. However, *Streptococcus* species have also been isolated in rare cases [2]. Infection frequently extends from the primary infection site, such as the pharynx or middle ear, to the lateral pharyngeal area, involving IJV, leading to septic thrombophlebitis and subsequent thrombus formation [3]. Lemierre syndrome is primarily diagnosed by the presence of IJV thrombosis and positive blood culture [1]. Intravenous antimicrobial should be initiated promptly and be continued for a duration of 4–6 weeks. The role of anticoagulants is unclear unless there is a thrombus propagation. The mortality rate was high in the pre-antimicrobial era but has decreased significantly since the introduction of antibiotics [4]. In conclusion, the clinician should be aware of this rare but fatal condition, and intravenous antimicrobial should be started without delay if clinically suspected.

## Ethical approval

Ethical approval was obtained from Medical Research Centre (MRC) in Hamad Medical Corporation (HMC).

## Consent

Written informed consent was obtained from the patient for publication of this case report and accompanying images.

## Funding

Open access publication funding was provided by Qatar National Library (QNL).

## Consent

Written informed consent was obtained from the patient for publication of this case report and accompanying images.

## CRediT authorship contribution statement

**Abdulrahman Al-Mashdali:** Conceptualization, Data curation, Writing – original draft, Writing – review & editing. **Akram Al-warqi:** Writing – original draft, Writing – review & editing.

## Declaration of Competing Interest

The authors report no declarations of interest.
